# Recent advances in understanding Alzheimer’s Disease: diagnosis and management strategies

**DOI:** 10.12703/r/12-24

**Published:** 2023-10-10

**Authors:** Anna Marin, Andrew E Budson

**Affiliations:** 1 Center for Translational Cognitive Neuroscience, Veterans Affairs Boston Healthcare System, Boston, MA, United States; 2 Alzheimer’s Disease Research Center, Boston University Aram V. Chobanian & Edward Avedisian School of Medicine, Boston, MA, United States; 3 Department of Behavioral Neuroscience, Boston University Aram V. Chobanian & Edward Avedisian School of Medicine, Boston, MA, United States

**Keywords:** Alzheimer’s Disease, diagnosis, strategies

## Abstract

As the rates of Alzheimer’s Disease (AD) increase in the world due to the aging of the population, research has made tremendous advances to target the two hallmark pathologies of AD: amyloid-β (Aβ) plaque deposition and neurofibrillary tangles of hyperphosphorylated tau. Here, we discuss recent advances in the clinical evaluation and management of AD, with a focus on new hypotheses related to the etiology of AD and new evidence related to AD-mimicking neurodegenerative diseases. Though recent clinical studies suggest anti-amyloid disease modifying agents may slow the progression of AD, there is currently no medication that stops it. Moreover, slowing the progression will result in more individuals in the mild cognitive impairment (MCI) and mild dementia stages of AD. Given this reality, we evaluate the development of non-pharmacological strategies to help sustain cognitive function and quality of life.

## Introduction

Numbers of individuals with Alzheimer’s disease (AD) and related dementias (ADRD) are growing rapidly. Given that age is the number one risk factor, we will soon be facing an unprecedented number of individuals with ADRD. Even current numbers are staggering, as an estimated 6 million Americans are currently diagnosed with AD, with direct and indirect costs for care estimated at $305 billion annually^1^. Compounding this issue, though recent clinical studies may slow the progression of AD, there is currently no medication that can stop the progression of the disease^2^. Nonetheless, important advances are being made in the clinical evaluation and management of AD. Current research efforts are exploring new causes at the root of AD pathology, are looking into ways to better identify other AD-mimicking neurodegenerative diseases and are developing targeted non-pharmacological strategies that can assist patients and their caregivers over the course of the disease. Understanding AD as not just a disorder of memory but a disorder of consciousness may be helpful in that regard^3^.

This review will attempt to summarize new advances in AD research as they relate to 1) clarification of AD etiology, 2) understanding of AD-mimicking diseases, and 3) development of strategies to target clinical symptoms and behavioral disturbances over the disease course.

## Current evidence and new hypotheses on AD neuropathology

In 2020, the FDA approved a new drug: Aducanumab. This new drug was heralded as one that would slow down cognitive decline through the removal of Aβ plaques^4,5^. However, despite the removal of amyloid plaques, the data presented to the US Food and Drug Administration (FDA) did not convincingly demonstrate a change in clinical function^6^. Moreover, there were significant side effects^7^, and it involves a high cost and a lifelong commitment to intravenous infusions^8^. New trials are continuing to study its clinical efficacy and its possible side effects^7,9,10^, and are also testing two new promising anti-amyloid medications, lecanemab^11^ and donanemab^12^. Lecanemab received accelerated FDA approval in January 2023, with full approval expected later in 2023. Donanemab is also expected to receive FDA approval soon.

Some researchers are taking a step back to re-evaluate the nature of Aβ under normal, non-pathologic conditions to inform the development of new treatment approaches. As such, in contrast to earlier perspectives that the role of Aβ is only disruptive to the brain, new research is exploring the innate role of Aβ as part of the immune response of the brain. At the core of this perspective is that the immune system is a main driver of Aβ clearance, and dysfunction in this system can lead to neuroinflammation, the brain’s response toward infection, and other causes of cell death^13,14^. In effect, Aβ acts as a peptide against bacteria, fungi, and viruses, which is beneficial in normal conditions but becomes detrimental in instances of chronic inflammation^15^. Several groups have advanced findings in support of the immune hypothesis in both human and rodent models. Namely, aging is shown to dysregulate the immune system, leading to increased susceptibility to infections and reduced healing abilities, resulting in the development of age-associated diseases related to inflammation^16^. A direct relationship has also been found between the lymphatic system, the removal of amyloid^17^, and cognitive impairment^18^. The worse the lymphatic system function is, the greater the amyloid buildup in the brain and the more cognitive impairment. Neuroinflammatory mechanisms causing AD result in amyloid misfolding^19^ and in the acceleration of Aβ deposition^20^. New treatments are being tested to target the processes of neuroinflammation^21^.

New evidence supports the perspective that tau neurofibrillary tangles are the primary cause of cognitive impairment in AD^22–24^, consistent with prior pathological studies^25^. Only recently could the regional distribution of tau tangles be measured prior to death. Today, thanks to the advent of tau-target PET tracers, the regional distribution of tau can be measured in living patients^26^. Thus, the amount of tau in the brain predicts future neurodegeneration^27^, and its location and pattern of spreading over time predicts the signs and symptoms of cognitive impairment^24,28,29^.

Thanks to tau imaging, research on the prevalence of patterns of AD neurodegeneration is quickly expanding. Earlier models argued that the majority of AD cases reflected one stereotyped pattern of tau progression. Recently, an international research collaboration led by Dr. Jacob Vogel found a different result. They longitudinally examined tau PET scans from 1,612 individuals with AD and developed a new model of tau spreading characterized by four distinct spatiotemporal pathways^30^. Although it has long been known that AD can present with frontal, visual, and language subtypes, in addition to the typical amnestic subtype^31^, and that these clinical patterns have been associated with 4 distinct spatiotemporal trajectories of tau pathology^30^, what they showed is that the prevalence of these 4 subtypes is roughly equivalent, ranging from 18 to 33%^30^. Thus, the amnestic subtype does not represent the majority of AD cases. Compared to other subtypes, those with the limbic (amnestic) subtype had better overall cognition but worse memory relative to their overall cognition; those with the MTL-sparing subtype (frontal) had relatively worse executive function; those with the lateral temporal subtype (language) had worse language and overall cognition with relatively better memory; and those with the posterior subtype (visual) were not significantly different from the other subtypes. Therefore, the single-patterned “typical” presentation of AD seems to have become obsolete, and tau imaging might reshape its management and treatment. Considering AD as a disorder of one or more aspects of consciousness—rather than memory—may be a more accurate and useful way of thinking about it^3^.

## Advances in the characterization of AD-mimicking neurodegenerative diseases

Alzheimer’s is the most common cause of dementia; however, other neurodegenerative diseases can mimic and/or co-occur, such as limbic-predominant age-related TDP-43 encephalopathy (LATE) and primary age-related tauopathy (PART). Many advances have been made in the characterization of these Alzheimer’s-like neurodegenerative diseases; however, increased awareness of these diseases is imperative to improve disease prognosis and symptom management.

### Limbic-predominant age-related TDP-43 encephalopathy (LATE)

LATE is the third most common cause of dementia, after AD and vascular disease^32^. In LATE, a specific phosphorylated protein (TDP-43) is found primarily in the amygdala (associated with emotions), the hippocampus (associated with episodic memory), and the anterior temporal lobes (associated with naming and semantic memory)^27,33^. LATE pathology often co-occurs with AD pathology and is found to contribute to some cognitive deficits observed in AD patients over 80^32^. LATE pathology may also occur by itself and, when AD biomarkers are not available, patients with LATE pathology alone are typically diagnosed with AD during life^27^. Studies suggest that individuals who only have LATE progress more slowly than individuals with AD, but when both pathologies co-occur, disease progression is faster than either one alone^32,33^. LATE can be easily mistaken for AD because it is also characterized by a progressive deficit in episodic memory and naming^34^; however, when evaluated in detail, the memory deficit in LATE is milder and isolated^35^. For example, verbal category fluency impairment, another hallmark cognitive symptom of AD, is not part of the clinical presentation of LATE^32,36^. Hippocampal sclerosis (dramatic hippocampal cell loss and atrophy) also has a similar clinical presentation to AD and is usually caused by LATE pathology. Older adults with hippocampal sclerosis caused by LATE tend to have severe impairment in episodic memory and can also present with a more global cognitive impairment that also affects semantic memory^37^.

### Primary age-related tauopathy (PART)

PART can also mimic AD: it is characterized by tau tangle pathology in the medial temporal lobe as in AD but lacks amyloid plaques. Although similar to AD, recent advances are showing that the type of tau found in PART and its genetic risk factors are not commonly associated with AD^33^. It also appears that PART may be inevitable with aging, as shown by the brains of centenarians at autopsy presenting tau pathology in the memory regions of the brain even if amyloid pathology was absent^33^.

Difficulties in diagnosing PART are often related to the fact that although older adults with PART complain of memory problems, they have very few deficits on cognitive testing. One study from the Mayo Clinic showed that their issues are actually related to an impairment of executive function (problem-solving, switching from one task to another), which is typically associated with the frontal part of the brain, not commonly linked with episodic memory^38^. Future research in the neuropsychological characterization of PART will be useful to improve its diagnostic accuracy and differentiation from AD.

### Future directions

LATE and PART pathologies may occur separately or in combination with AD. Future research and clinical efforts should be aware of the differences between LATE, PART, and AD to improve disease prognosis and ensure that treatments are tailored toward each pathology. These efforts will improve clinical care and support current trials aiming to find disease-modifying treatments specific for AD.

## Non-pharmacological strategies to target early and late stages of AD

Many researchers are advocating for the use of non-pharmacological strategies to ameliorate the quality of daily life for patients and their caregivers^39^. Current research efforts on non-pharmacological strategies focus on the clinical assessment of patients with AD and on the management of the cognitive and behavioral symptoms at the early and later stages of the disease.

### Clinical Assessment

To improve the effectiveness of non-pharmacological strategies, researchers are trying to better understand the many facets that characterize the cognitive deficits in AD^40^. One of the most commonly acknowledged clinical symptoms of AD is forgetting; however, another type of memory error, known as false memories, also has an important clinical relevance and should not be ignored^41^. False memories occur when things are remembered as being experienced when, in fact, they were not. Through the development of a new questionnaire to measure the frequency of false memories from the perspective of clinicians and family members, researchers have shown that false memories are more prevalent in the everyday life of AD patients compared to healthy older adults and that in AD patients they are almost as prevalent as the symptom of forgetting^41^. These findings underscore the role of false memories in disease diagnosis and management. Future clinical trials should measure false memories together with other variables of memory and daily life functioning to capture a more accurate picture of the cognitive signs and symptoms that patients with AD and their caregivers experience.

Improvements in the diagnosis and characterization of AD are critical to enhancing the effectiveness of non-pharmacological interventions. Current work is exploring the utility of electroencephalography (EEG), and specifically event-related potentials (ERPs), as a diagnostic screening measure for memory impairment and as a tool to monitor disease progression. EEG/ERPs are a time-locked measure of brain activity that reflects both sensory and cognitive responses to stimuli. Researchers are showing that EEG/ERPs represent an accessible tool to help diagnose AD early and to examine changes over the course of the disease^42–45^. Future work should investigate how EEG/ERPs may be employed to assist in diagnosis and to monitor changes in cognitive function over time in patients with AD.

### Clinical management

In light of the greater susceptibility to memory errors compared to the average older population, recent work is evaluating cognitive strategies to aid memory accuracy in patients with AD. At the earlier stages of the disease, two memory strategies, especially when used together, help decrease the occurrence of false memories^46^. The first strategy is designed to help patients learn new information and recommends generating one or more unique qualities of the new information being learned (e.g., how does it relate to a personal experience?). The second strategy is designed to help reduce the recall of false information and recommends endorsing a memory as true only if one is quite sure of it to reduce memory errors. Together, the use of these memory strategies may improve memory quality in patients in the mild stages of the disease, which in turn allows these patients to function more independently and maintain a good quality of life for a longer period of time^41^.

At later stages of the disease, behavioral disturbances, such as agitation and aggression, become a critical source of distress for patients and their caregivers. Easy-to-use, evidence-based behavioral approaches, such as the Describe, Investigate, Create, and Evaluate (DICE) approach, can help caregivers identify and manage behavioral symptoms of the middle and late stages of AD dementia while supporting their own health and wellbeing^47–50^. These new disease management tools are also useful when combined with pharmacological treatments and can be tailored to the patients’ and caregivers’ individualized needs^50^. Caregivers are trained to apply behavioral approaches such as the 4Rs: Reassure, Reconsider, Redirect, and Relax^51^. These general non-pharmacologic approaches can help caregivers and patients while preventing the overuse of medications.

Music therapy is a specific type of behavioral strategy that is increasingly used with AD patients. It has been found to help improve patients’ cognitive and behavioral symptoms as well as caregivers’ moods^52–54^. In the context of long-term residential units, the use of music therapy protocols also enhances the staff’s awareness of the importance of psychosocial strategies for patients’ wellbeing^55^. Other advances in behavioral strategies focus on the impact of environmental factors, such as access and exposure to green spaces, on mood and cognitive function in AD patients. Green spaces have been found to have both direct and indirect influences on cognitive function^56^. Tailored psychosocial and environmental-exposure protocols can help improve quality of life in patients with AD and their caregivers^42^.

Although there are still ongoing challenges to standardize these behavioral protocols on a day-to-day basis for individual patients, new studies are showing that creating space for non-pharmacological strategies can have a real positive outcome on patients’ function and wellbeing^57^. Families and professional caregivers need to become more aware of the importance of these strategies, and clinical leaders should increase the engagement of staff in these behavioral treatment protocols.

## Summary

The number of patients with AD dementia is rapidly increasing, while the development of effective disease-modifying treatments is still ongoing. Though recent clinical trials testing the effectiveness of anti-Aβ medications to slow clinical deterioration appear to be modestly effective, current research is exploring other factors driving disease pathophysiology, with a specific focus on the role of neuroinflammation. Thanks to the recent advances in the techniques to detect tau biomarkers in vivo, the spreading pattern of tau within the brain can predict the resulting cognitive symptoms, and help clinicians and researchers understand that the majority of AD patients do not present with the stereotypic amnestic subtype. The high prevalence of two AD-mimicking neurodegenerative diseases, LATE and PART, is now better recognized. New work is also supporting the utility of non-pharmacological strategies to aid cognitive function early and to better manage behavioral symptoms in later stages of AD. More resources are necessary to implement behavioral strategies in a systematic and reproducible manner, both to increase true memories and to reduce false memories. Given the rising prevalence and mortality of AD coupled with the growing burden on the caregiver’s health and healthcare costs, the medical community should promote the development of effective and realistic ways to diagnose, manage, and successfully treat this neurodegenerative disease, with the central goal to sustain patients’ quality of life and the wellbeing of those caring for them.
